# Envelope solitary waves exist and collide head-on without phase shift in a dusty plasma

**DOI:** 10.1038/srep14239

**Published:** 2015-09-18

**Authors:** Heng Zhang, Xin Qi, Wen-Shan Duan, Lei Yang

**Affiliations:** 1 College of Physics and Electronic Engineering and Joint Laboratory of Atomic and Molecular Physics of NWNU & IMP CAS, Northwest Normal University, Lanzhou 730070 and Institute of Modern Physics, Chinese Academy of Sciences, Lanzhou 730000, China; 2 Department of Physics, Lanzhou University, Lanzhou 730000, China

## Abstract

The rarefactive KdV solitary waves in a dusty plasma have been extensively studied analytically and found experimentally in the previous works. Though the envelope solitary wave described by a nonlinear Schrödinger equation (NLSE) has been proposed by using the reductive perturbation method, it is first verified by using the particle-in-cell (PIC) numerical method in this paper. Surprisingly, there is no phase shift after the head on collision between two envelope solitary waves, while it is sure that there are phase shifts of two colliding KdV solitary waves after head on collision.

Dusty plasmas have been extensively studied because of their relevance in many space and technological applications^1^,^2^, as well as in the confined fusion system^3^,^4^. Dusty plasma supports a variety of collective modes and nonlinear coherent structures, therefore, there are many research works on both the linear and nonlinear waves for dusty plasmas^5^,^6^,^7^,^8^,^9^,^10^,^11^,^12^,^13^,^14^,^15^.

The rarefactive nonlinear waves in a dusty plasma have been extensively studied analytically during the past years by using the traditional reductive perturbation method^16^ and verified by the experiments^13^,^14^,^15^. Recently, the application scope of the reductive perturbation method in a dusty plasma has been given by using the PIC numerical method^17^. On the other hand, the compressed nonlinear waves in a electron-ion (EI) plasma have also been found by using the reductive perturbation method analytically and verified by PIC numerical simulation^18^. The remarkable distinction between a EI plasma and a dusty plasma is that one is the compressed wave and the other is the rarefactive one. However, the envelope solitary wave is neither compressed nor rarefactive which is described by the NLSE obtained by the reductive perturbation method^19^,^20^. This kind of nonlinear waves have been studied previously. For example, Admin and Shukla *et al.*^21^ studied the modulational instability of the dust acoustic waves and the dust-ion-waves. Ghosh *et al.*^22^ studied the effects of the dust charge fluctuations of the low-frequency wave modulation. However, whether the envelope solitary wave really existence in a dusty plasma is still remain unsolved since it is not verified by either the experiments or the numerical simulation until now. The objective and implications of the present results is as follows. First one is to verify the existence of the envelope solitary wave in dusty plasmas by using the PIC method. Second, the application scope of the traditional reductive perturbation method to obtain the NLSE will be checked. Third, the characters of the envelope solitary wave can be applied to the space and technological applications, as well as in the magnetically confined fusion system.

For simplicity, we neglect the effect of the dust charge fluctuation in the present paper and assume that the dust charge is a constant since we want to know if the envelope solitary wave really exist in a dusty plasma by using the PIC numerical method. Though if the effect of the dust charge fluctuation is considered, the damped NLSE can be obtained^21^. The numerical simulation by PIC method may be a future work to investigate the effect of the dust charge fluctuation on the envelope solitary wave, i.e., how the dust charge fluctuation damp the envelope solitary wave.

By using the PIC method, we first verify the envelop solitary wave which exists in a dusty plasma. Then the application scope of the analytical solution described by NLSE is given. The head on collision between two envelop solitary waves is also simulated by using the PIC method. Surprisingly, it is found that there is no phase delay during the collision between two envelop solitary waves which is different from that between two KdV solitary waves in which there are phase delays for both colliding solitary waves^23^. However, it is noted that the envelope solitary wave can be considered as envelope soliton since it will remain its waveform and the propagation velocity after the head on collision.

## Results

### Analytical solution of an envelope solitary wave by using the perturbation method

The propagation of an envelop solitary wave and the head on collision between them are studied by one dimensional (1D) PIC method in infinite background plasma. Before our simulation, we first give an analytical solution of an envelop solitary wave by using the reductive perturbation method.

The one-dimensional dimensionless equations of the motion of a dusty plasma are^23^


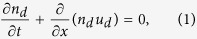



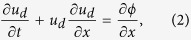



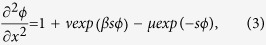


where *β* = *T*_*i*_/*T*_*e*_ is the ratio of the ion and electron temperatures, *s* = 1/(*μ* + *νβ*), *μ* and *ν* are the normalized ion and electron number densities, respectively. *n*_*d*_ and *u*_*d*_ refer to the density, the velocity of the dust grains respectively. *ϕ* is the electrostatic potential.

The spatial coordinate *x*, the time *t*, the velocity *u*_*d*_ and the electrostatic potential *ϕ* are normalized by the Debye length *λ*_*D*_ = (*T*_*eff*_/4*πZ*_*d*_*n*_*d*0_*e*^2^)^1/2^, the inverse of effective dust plasma frequency 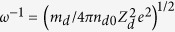, the dust acoustic speed *C*_*d*_ = (*Z*_*d*_*T*_*eff*_/*m*_*d*_)^1/2^ and *T*_*eff*_/*e*, respectively, where the effective temperature is defined as 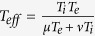.

In order to study envelope solitary wave in a dusty plasma, we introduce the following stretched coordinates according to the traditional perturbation method: *ξ* = *ε*(*x* − *u*_*s*_*t*), *τ* = *ε*^2^*t*. All the physical quantities are expanded as follows: 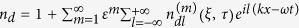, 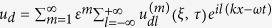, 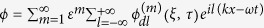. By substituting these expansions into the equations of motion, we have the following results: 
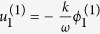
, 
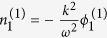
, the dispersion relation: 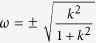, the group velocity: 
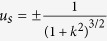
, and finally the NLSE^21^,^24^


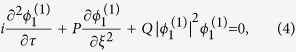


where


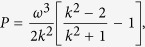



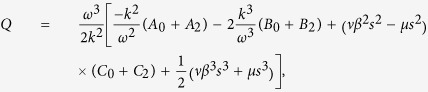


and


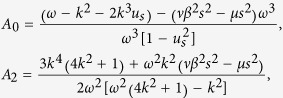



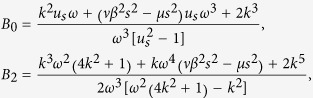



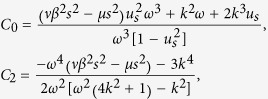


where *P* < 0, *Q* < 0.

One of the well known solution of NLSE of Eq. (4) is an envelope solitary wave as follows^21^,^24^


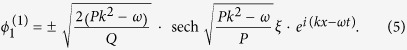


If the signs of both *ω* and *u*_*s*_ are positive, the envelope solitary wave propagates in the positive *x* direction. Otherwise, it propagates in the negative *x* direction.

### PIC simulation results

**Figure 1:**
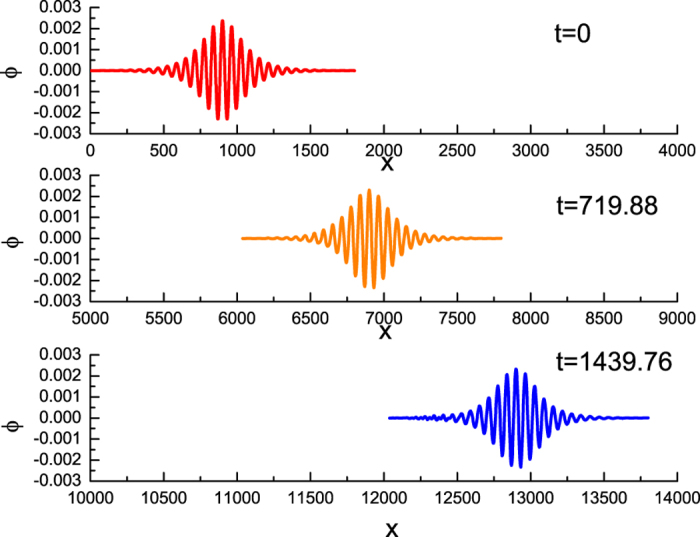
The PIC simulation results of the evolution of an envelope solitary wave at the different times where *ε* = 0.01, *k* = 0.1, *μ* = 1.1, *ν* = 0.1, *β* = 0.1.

**Figure 2:**
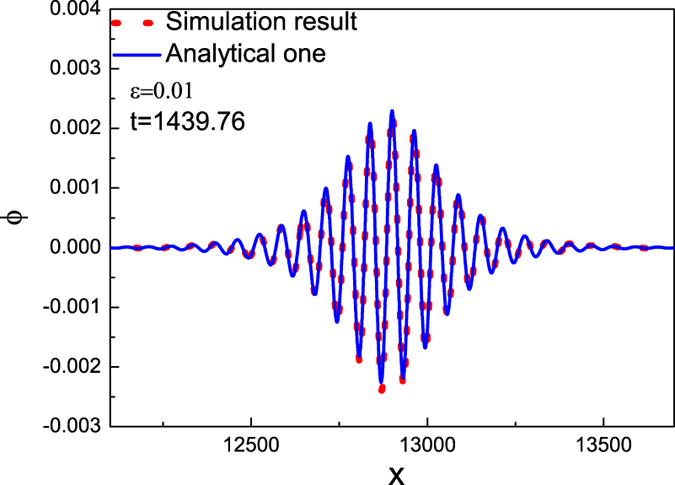
The comparisons between PIC simulation results and the analytical ones with the same initial conditions, where *ε* = 0.01, *k* = 0.1, *μ* = 1.1, *ν* = 0.1, *β* = 0.1.

The PIC simulation results are given in Fig. 1 at different times. It is observed that the waveform of this fluctuation (envelop solitary wave) remain unchanged and its propagation speed is a constant. We compare our numerical results with the analytical one of Eq. (5) and find a good agreements between them, as shown in Fig. 2.

**Figure 3:**
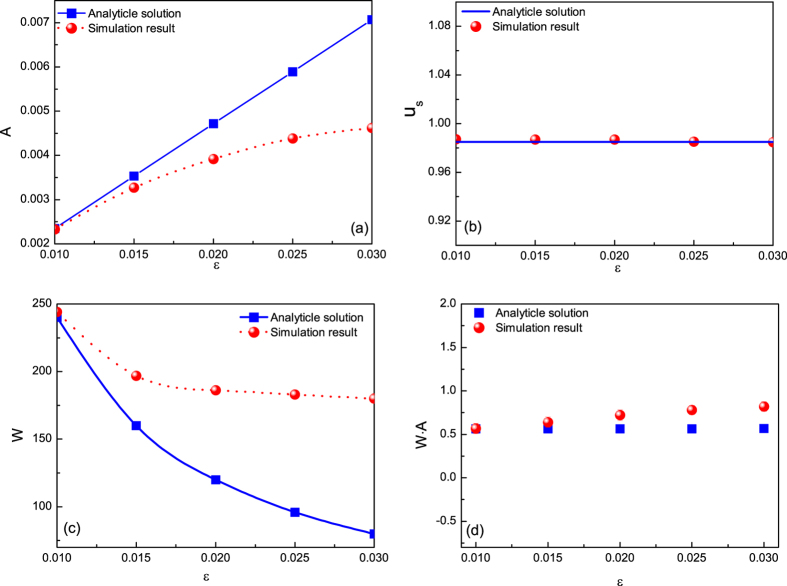
Comparisons between the simulation results and the analytical ones. (**a**) Dependence of the amplitude of the envelope solitary wave on the parameter *ε*. (**b**) Dependence of the propagation speed of the envelope solitary wave on the parameter *ε*. (**c**) Dependence of the width of of the envelope solitary wave on the parameter *ε*. (**d**) Dependence of the product of amplitude and width of the envelope solitary wave on parameter *ε*.

To gain more insight into this envelope solitary wave, more PIC results are given for different values of *ε*. The dependence of the amplitude, the propagation velocity and the width of the envelope solitary wave on the parameter *ε* are shown in Fig. 3. It is noted that the envelope solitary wave can steadily propagate in the dusty plasma if the parameter *ε* is small enough. The good agreement between the PIC results and the analytical ones are observed, which indicates that the analytical result obtained by the reductive perturbation method is reliable if the amplitude of the envelope solitary wave is small enough, or *ε* < ε^*^. It is noted that ε^*^ ≈ 0.02. The obvious differences between two are observed if *ε* > ε^*^. It indicates that the application scope of the perturbation method is within the range *ε* < ε^*^. However, it is noted that the propagation velocity is independent of the parameter *ε*, i.e., the group velocity is a constant, which is also in agreements with the analytical one. It is also noted from Fig. 3(d) that the product of the amplitude and the width of the envelope solitary wave is a constant, while the analytical result which can be obtained from Eq. (5) is 
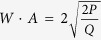
. Both are in good agreements. However, it is reported experimentally that the product of the amplitude and the square of its width of the KdV solitary wave is a constant^14^ which is different from that of the envelope solitary wave.

### Head on collision between two envelope solitary waves

Let us see what is observed if two opposite propagating envelope solitary waves collide. It is well known that the head on collision between two opposite propagating KdV solitary waves can pass through each other as if without suffering any interaction except for a slight phase shift in their positions. It has been extensively studied by using the extended Poincare-Lighthill-Kuo (PLK) method analytically^20^,^25^,^26^,^27^,^28^ and verified in the experiments^19^. Recently the application scope of the PLK method on the colliding of two KdV solitary waves is studied by using the PIC method^23^. However, the head on collision between two envelope solitary waves has not been studied until now. We now study the head on collision between two envelope solitary waves by using the PIC method.

**Figure 4:**
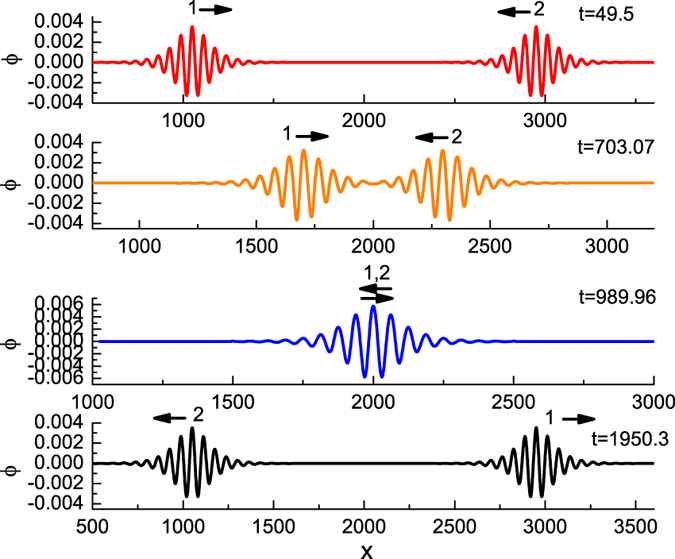
The evolution of head on collision between two envelope solitary waves at different time, where *ε*_1_ = *ε*_2_ = 0.015, *k* = 0.1, *ν* = 0.1, *μ* = 1.1 and *β* = 0.1.

We assume that there are two opposite propagating envelope solitary waves. One is propagating in the positive *x* direction and the other is in the negative *x* direction. Initially, both solitary waves are far apart. After some time, they interact, collide, and then depart. The colliding process are shown in Fig. 4. It is observed that the waveforms of both waves remain unchanged after the collision. It seems that they are envelope solitons. However, a remarkable phenomena is noted that there is no phase shift for both colliding waves. More numerical results have verified this conclusion. As well known, however, there are phase shifts after head on collision for the KdV solitary waves^23^. The result is much interesting because there are obvious distinction of the head on collision between the KdV solitary waves and the envelope waves. There is a phase shift after collision for KdV solitary waves, while there is no phase shift for two envelope solitary waves.

**Figure 5:**
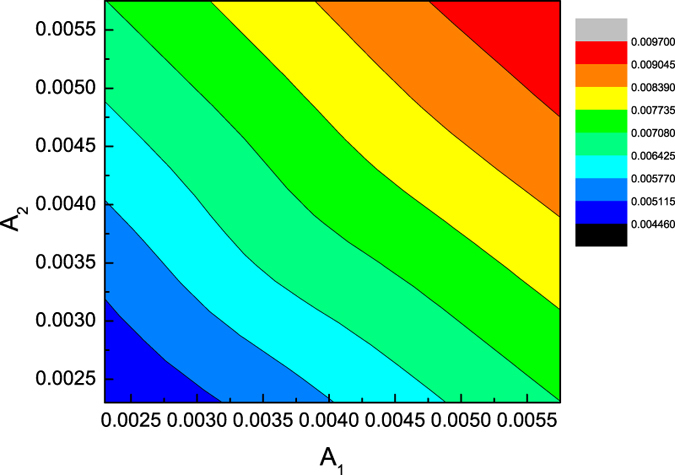
The dependence of the maximum amplitude in the colliding process on both amplitudes (*A*_1_, *A*_2_) of two colliding solitary waves 1 and 2.

**Figure 6:**
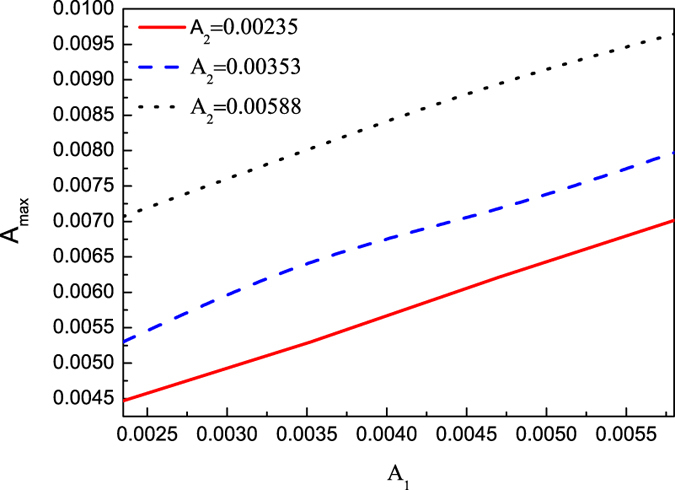
The dependence of the maximum amplitude in the colliding process on amplitude *A*_1_ for different *A*_2_.

Furthermore, the dependence of the maximum amplitudes of the solitary waves during the colliding process on both the initial amplitudes of colliding solitary waves is shown in Fig. 5. It is more clearly observed that the maximum amplitude in the colliding process increases as the two amplitudes of the colliding solitary waves increase in Fig. 6. It seems that the maximum amplitude in the colliding process is less than the sum of two amplitudes of envelope solitary waves.

## Discussion

By using the PIC method, we have verified that the envelope solitary wave can exist in a dusty plasmas. The application scope of the reductive perturbation method to derive a envelope solitary wave is given. The head on collision between two envelope solitary waves is implemented in a dusty plasma by using the PIC simulation method. The interesting results are that there is no phase shift after the collision between two envelope solitary waves which is different from that between two KdV solitary waves. Moreover, the PIC simulation method can be used to verify that whether a series of solitary wave solutions obtained by perturbation method really exist in either dusty plasma or EI plasma.

## Methods

**Figure 7:**
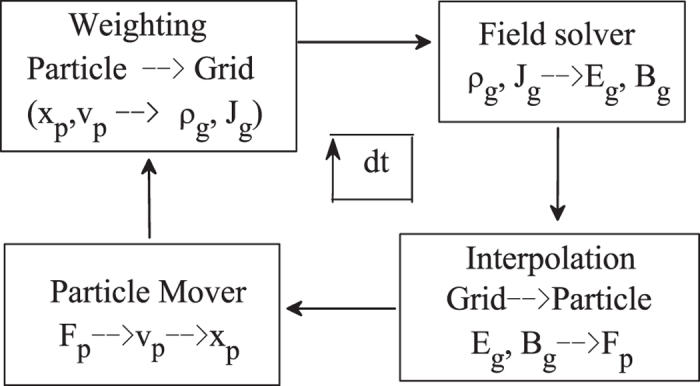
The comparisons between the numerical results and the analytical ones.

Numerical experiment is performed by using the one-dimensional PIC simulation method to study the formation and the propagation of an envelope solitary wave in a dusty plasma in the present work. During the simulation, The dust particles are represented as kinetic particles, while ions and electrons are modeled as Boltzmann distributed background. As well known, the real systems always contain very large amount of particles. In order to make simulations efficient or at least possible, so-called super-particles(SPs) are used. Each SP has a weight factor S specifying the number of real particles contained. Therefore, the equation of motion of the system is the Newton’s equation as follows


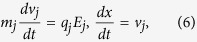


where *m*_*j*_, *q*_*j*_, *x*_*j*_ are the mass, charge and position of the *j*th SP, respectively. *E*_*j*_ is the electric field at the position of the *j*th SP. As the dust particles follow their trajectories, they continually exchange information with the background grid. Each dust particle contributes its charge to the corners of its instantaneous host cell. Therefore, the simulation region is divided to contain several grid cells during the PIC simulation. At each time step, the velocities, the positions of SPs are weighted to all the grids to calculate the charge density *ρ*_*g*_ (or electric current density *J*_*g*_). Once *ρ*_*g*_ obtained the Maxwell’s equations (electromagnetic model) or Poisson-Boltzmann equation (electrostatic model) will be solved numerically to derive the value of *E* at each grid. In electrostatic model, *B*_*g*_ = 0. Then the field imposed on each SP can be worked out and each SP will be driven by electric field according to Eq. (6), which will be solved numerically via the leap-frog algorithm. At last, the new positions and velocities are obtained, the procedure come to repeat until the simulation completed. The summary of a computational cycle of the PIC method is shown in Fig 7.

In the PIC simulation, initial conditions are chosen from the analytical solution expressed by Eq. (5) at a certain time. The initial values of the number density and the velocity of the dust particles are: 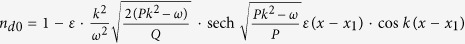, and 
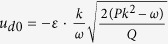

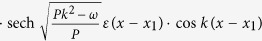
, respectively. Space coordinate *x*, time *t*, grid size Δ*x*, time step Δ*t*, number density *n*_*d*_ and the self-consistent potential *ϕ* are all dimensionless parameters. Based on the limitations attached to the PIC method, the simulation parameters are chosen as follows: Δ*x* = 0.3, Δ*t* = Δ*x*/100, the number of grid cells is *N*_*x*_ = 6000 and the number of super particles contained per cell is 50, the total length of *x*-axis is *LX* = Δ*xN*_*x*_. *ε* = 0.01, *k* = 0.1, *μ* = 1.1, *ν* = 0.1, *β* = 0.1, *ω* = 0.1, *u*_*s*_ = 0.985, *x*_1_ = *LX*/4. This initial disturbance will evolve as the time increases. The boundary conditions along the *x*-axis are periodic. To avoid wave reflection at the boundary, a frame moving with envelope solitary wave is introduced, so that the envelope solitary wave remains away from the boundary. At the beginning of the simulation, the SPs representing dust particles are distributed uniformly in the whole simulation region. In simulations, an envelope solitary wave is initially given which propagates in the positive *x* direction in an infinite background plasma.

In process of head-on collision between two envelope solitary waves, we assume that there are two opposite propagating envelope solitary waves. One is propagating in the positive *x* direction and the other is in the negative *x* direction. The initial number density and the velocity of the dust particles are: 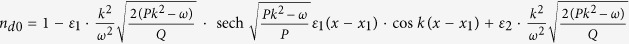

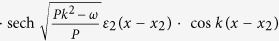
, and 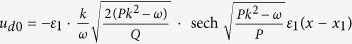

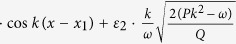

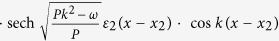
, respectively. Initially, both solitary waves are far apart. The initial conditions are chosen from the analytical results of Eq. (5). The parameters of two colliding waves ar *ε*_1_ = *ε*_2_ = 0.015, *k* = 0.1, *μ* = 1.1, *ν* = 0.1, *β* = 0.1, *x*_1_ = *LX*/4, *x*_2_ = 3*LX*/4, Δ*x* = 0.3, Δ*t* = Δ*x*/100, *N*_*x*_ = 20000, while *ω* = ±0.1, *u*_*s*_ = ±0.985 and the number of super particles contained per cell is 100, where the positive sign stands for the solitary wave propagating in the positive *x* direction, while the negative one represents that in the negative *x* direction.

## Additional Information

**How to cite this article**: Zhang, H. *et al.* Envelope solitary waves exist and collide head-on without phase shift in a dusty plasma. *Sci. Rep.* **5**, 14239; doi: 10.1038/srep14239 (2015).
